# Copper(II)-Mediated Iodination of 1-Nitroso-2-naphthol

**DOI:** 10.3390/molecules26185708

**Published:** 2021-09-21

**Authors:** Zarina M. Efimenko, Anton V. Rozhkov, Vitalii V. Suslonov, Maxim L. Kuznetsov, Vadim Yu. Kukushkin, Nadezhda A. Bokach

**Affiliations:** 1 Institute of Chemistry, Saint Petersburg State University, Universitetskaya Nab. 7/9, 199034 Saint Petersburg, Russia; bicbaevazarina@yandex.ru (Z.M.E.); iomcrozhkov@gmail.com (A.V.R.); v.suslonov@spbu.ru (V.V.S.); max@mail.ist.utl.pt (M.L.K.); v.kukushkin@spbu.ru (V.Y.K.); 2 Centro de Química Estrutural, Instituto Superior Técnico, Universidade de Lisboa, 1049-001 Lisbon, Portugal; 3 Institute of Chemistry and Pharmaceutical Technologies, Altai State University, 656049 Barnaul, Russia

**Keywords:** metal-mediated reaction, iodination, copper(II) complexes, nitrosonaphthol, halogen bonding

## Abstract

The 3-Iodo-1-nitrosonaphthalene-2-ol (I-NON) was obtained by the copper(II)-mediated iodination of 1-nitroso-2-naphthol (NON). The suitable reactants and optimized reaction conditions, providing 94% NMR yield of I-NON, included the usage of Cu(OAc)_2_·H_2_O and 1:2:8 Cu^II^/NON/I_2_ molar ratio between the reactants. The obtained I-NON was characterized by elemental analyses (C, H, N), high-resolution ESI^+^-MS, ^1^H and ^13^C{^1^H} NMR, FTIR, UV-vis spectroscopy, TGA, and X-ray crystallography (XRD). The copper(II) complexes bearing deprotonated I-NON were prepared as follows: *cis*-[Cu(I-NON–H)(I-NON)](I_3_) (**1**) was obtained by the reaction between Cu(NON-H)_2_ and I_2_ in CHCl_3_/MeOH, while *trans*-[Cu(I-NON–H)_2_] (**2**) was synthesized from I-NON and Cu(OAc)_2_ in MeOH. Crystals of *trans*-[Cu(I-NON–H)_2_(THF)_2_] (**3**) and *trans*-[Cu(I-NON–H)_2_(Py)_2_] (**4**) were precipitated from solutions of **2** in CHCl_3_/THF and Py/CHCl_3_/MeOH mixtures, respectively. The structures of **1** and **3**–**4** were additionally verified by X-ray crystallography. The characteristic feature of the structures of **1** and **3** is the presence of intermolecular halogen bonds with the involvement of the iodine center of the metal-bound deprotonated I-NON. The nature of the I···I and I···O contacts in the structures of **1** and **3**, correspondingly, were studied theoretically at the DFT (PBE0-D3BJ) level using the QTAIM, ESP, ELF, NBO, and IGM methods.

## 1. Introduction

Nitrosonaphthols, typically existing in the quinone monoxime tautomeric form (see ESI, Scheme S1) [1], represent a group of compounds that found a wide range of useful applications. These species have been applied as precursors in organic synthesis [2], they were used for the analytical determination of bioorganic species [3,4], and they also exhibit some biological activity modes (e.g., carcinogenic) as iminoxyl and hydronitroxide radical source [5]. Nitrosonaphthols with *ortho*-positioned functional groups, i.e., NO(H) and O(H), exhibit chelating properties and they found application as analytical reagents for determination [6,7,8], extraction [7,8,9], and sorption [10] of metal ions and also can be applied as sequestering agents for nanoparticle preparation [11]. The *o-*nitrosonaphthols were also used for the preparation of immobilized reagents for optical metal ion sensing [12] and polymer-supported catalysts [13].

A search of synthetically significant routes for the modification and functionalization of nitrosonaphthols could be an important task for the improvement of the analytical properties of these and structurally related species. In particular, initial modification of nitrosonaphthols can include introduction of a halogen atom, e.g., by direct halogenation. The direct chlorination and bromination of 1-nitroso-2-naphthol by the corresponding dihalogens to afford 3-halo-1-nitroso-2-naphthols have been reported [14,15], while the iodination is a more complicated task and the iodinated product was obtained only by the exchange reaction of the corresponding brominated derivative [15]. Indeed, the halogenation of aromatic species by X_2_ strongly depends on the identity of a halogen and, in the case of less reactive I_2_, this reaction commonly requires Lewis acid catalysis, e.g., application of copper(II) salts, and it was conducted mainly for electron-rich arenes [16,17,18]. In this work, we report on a novel metal-mediated reaction that proceeds in the system Cu^II^/1-nitroso-2-naphthol/I_2_ and leads, after liberation of the ligand, to uncomplexed 3-iodo-1-nitrosonaphthalene-2-ol. We also succeeded in obtaining several copper(II) complexes with the iodinated product, and all our results and findings are detailed in sections that follow.

## 2. Results

### 2.1. Synthesis of 3-Iodo-1-nitrosonaphthalen-2-ol

**Scheme 1:**
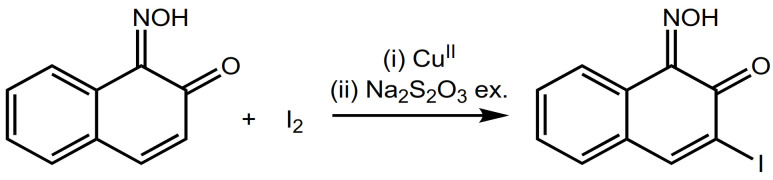
Synthesis of I-NON via Cu^II^-mediated iodination of NON.

The 3-Iodo-1-nitrosonaphthalene-2-ol (I-NON) was obtained by the copper(II)-mediated iodination of 1-nitroso-2-naphthol (NON). Our initial experiments demonstrated that the copper complex [Cu(NON–H)_2_] reacted with 8 equivs of molecular iodine in MeOH to form the [Cu(I-NON–H)(I-NON)](I_3_) (**1**) complex bearing the iodinated ligands (for detailed characterization and relevant discussion, see below). We applied this reaction as a starting point to develop a synthetic route to I-NON (Scheme 1). On the one hand, I-NON, as a representative of the group of nitrosonaphthols, can be useful as a chelator for transition metal complexes and this sequestering can be applied for analytic or extraction purposes [19,20]; on the other hand, the iodine functionality in I-NON, acting as a halogen bond (HaB) donor, might be useful for HaB-based crystal engineering [21,22,23,24,25].

Our procedure consists, on the first step, in one-pot reaction of NON with a copper(II) salt and I_2_ followed by, on the second step, the liberation of I-NON by the treatment of the reaction mixture with an excess Na_2_S_2_O_3_. Aiming to develop the two-step synthetic procedure to I-NON, we performed the optimization of the reaction conditions (Table 1) by varying copper(II) and some other metal salts, load of copper(II) salts, and variation of molar ratios between the reactants. We tested several copper(II) sources (Table 1, entries 1–5) for the system Cu^II^/NON/I_2_ (1:2:2 molar ratio) and found that the application of CuCl_2_·2H_2_O gave the highest yield of I-NON (38%), while the involvement of Cu(OAc)_2_·H_2_O produced the best ratio between the product and the unreacted starting materials (2:1). Therefore, for further optimization, we chose Cu(OAc)_2_·H_2_O. Variation of the I_2_ load (Table 1, entries 5–8) revealed that the highest yield was achieved with 1:4 NON/I_2_ molar ratio. We also attempted the reaction under the catalytic conditions (Table 1, entries 5 and 9, 7 and 10), but observed the I-NON yield drop on decreasing the load of Cu(OAc)_2_·H_2_O. This experiment indicates that the reaction required the stochiometric amount of copper(II). Other tested metal salts were less effective (Table 1, entry 14) or totally ineffective (Table 1, entries 11–13) in the reaction. Notably, the iodination of NON can proceed under metal-free conditions (Table 1, entry 15), but, in contrast to the optimized Cu^II^-mediated reaction, this synthesis gave only ca. 24% conversion to I-NON. An increase of the reaction time to 3 d did not affect the conversion of the starting materials. In summary, the results of these tests demonstrated that the optimal conditions for this copper(II)-involving reaction included the usage of Cu(OAc)_2_·H_2_O and 1:2:8 Cu^II^/NON/I_2_ molar ratios between the reactants. Although the reaction, as we found, could be carried out without the metal source (ca. 25% conversion, as verified by GC-MS monitoring; the isolation was not performed), the employment of copper(II) led to *nearly quantitative* conversion of NON to I-NON (94%; entry 8).

Based on all these observations, one can conclude that the iodination of NON proceeded in the coordination sphere of Cu^II^, while the optimal reactant ratio Cu^II^/NON (1:2) corresponded to the coordination of NON to give {Cu(NON–H)_2_} species, which then converted to the iodination product, bearing the {Cu(I-NON–H)_2_} moiety. Therefore, Na_2_S_2_O_3_ on the second step of the synthetic scheme can play the dual role: It was required for the neutralization of the excess of I_2_ and, simultaneously, it liberated I-NON from the formed complex via the Cu^II^-to-Cu^I^ reduction, followed by the complexation of the latter with I^–^ and the precipitation of CuI.

After the optimization, we also attempted to extend this copper(II)-mediated reaction to other substrates, such as 2-nitroso-1-naphthol or 1,2-nitrosophenol; however, only mixtures of yet unidentified products were obtained and any iodination product was not detected by ^1^H NMR or HRESI^+^-MS.

**Figure 1:**
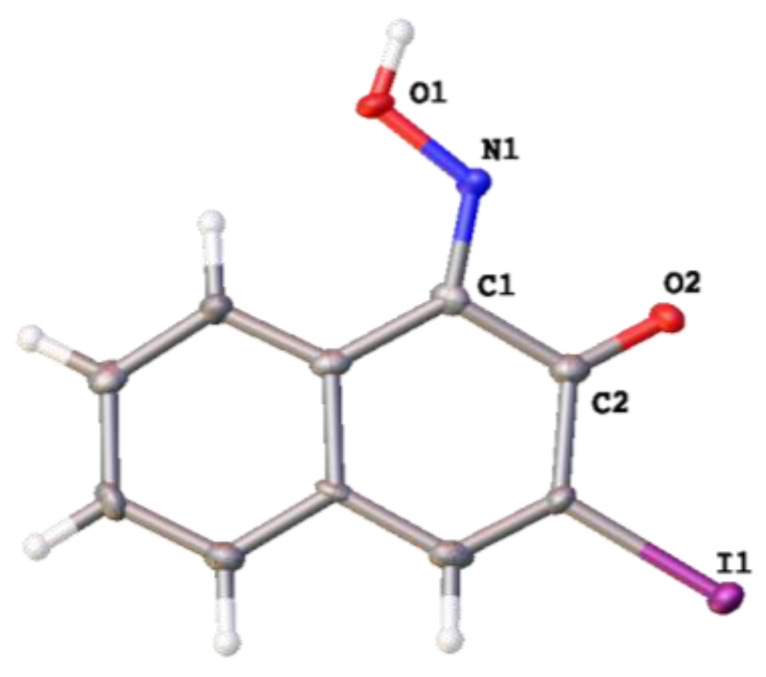
View of the molecular structure of I-NON obtained from XRD study. Nonhydrogen atoms are given as probability ellipsoids of atomic displacements (*p* = 0.5).

The structure of I-NON was studied by single-crystal X-ray diffractometry (XRD, Figure 1). Inspection of bond lengths’ distribution (N1–O1 1.368(11), N1–C1 1.296(13), C2–O2 1.225(13) Å) confirmed that I-NON existed in the quinone monoxime form in a solid state, similarly to solid NON [26]. Each of two neighboring molecules of I-NON gave dimers held by two intermolecular hydrogen bonds O1–H1···N1 (O1–N1 2.781(14) Å, ∠O1–H1–N1 132.9(6)°) (Figure S18, **ESI**). Molecules were also connected via Type I [21,27] I···I contacts (I1I1 3.7550(16) Å, ∠I1–I1–C3 162.7(3)°). For characterization of I-NON, see the ESI.

Thus, we developed a simple and facile approach to I-NON starting from NON. We found only one study focused on the synthesis of I-NON. The latter included the reaction of 3-bromo-1-nitrosonaphthalen-2-ol (Br-NON) with excess KI to give I-NON in 60% yield [28]. In turn, Br-NON can be obtained by the bromination of NON with Br_2_ [29]. Our procedure gave a significantly higher yield of I-NON (94%) and did not require the usage of the harmful Br_2_.

We verified the role of metal center control on the reactivity of the deprotonated NON *ligand* as compared to *uncomplexed* NON, which is, at least, dual. Firstly, it was protecting the oxime nitrogen from the well-known oxidative deoximation (ref. [30] and references therein) and, secondly, the copper site stabilized the oxo(1–)-substituent in the benzene ring directing the iodination in the *ortho-*position. Conventional activation of I_2_ by a Lewis acid should also be taken into account.

### 2.2. Copper(II) Complexes Featuring 3-Iodo-1-nitrosonaphthalen-2-ol

I-NON, as the structural analog of NON, can be also applied as a ligand for metal binding. We studied the ligand properties of I-NON and thus synthesized two I-NON-based Cu^II^ complexes. The complex *trans*-[Cu(I-NON–H)_2_] (**2**) was obtained by the reaction of I-NON with Cu(OAc)_2_·H_2_O in MeOH and it was isolated as a black solid, while *cis*-[Cu(I-NON–H)(I-NON)](I_3_) (**1**) was generated by the treatment of [Cu(NON–H)_2_] with 8-fold excess of I_2_ in a CHCl_3_/MeOH solution at RT. For details of characterization of **1** and **2,** see ESI.

**Figure 2:**
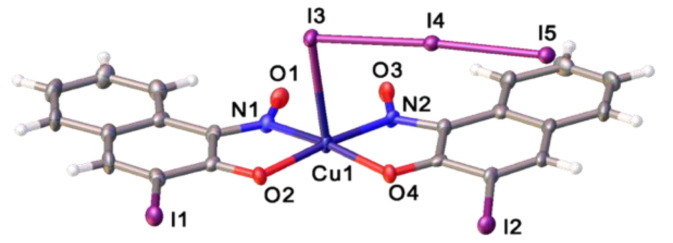
View of the molecular structure of **1** obtained from XRD study. Nonhydrogen atoms are given as probability ellipsoids of atomic displacements (*p* = 0.5).

**Figure 3:**
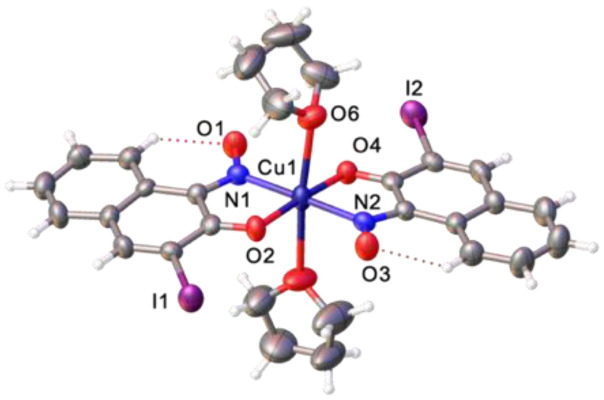
View of the molecular structure of **3** obtained from XRD study. Only one from two symmetrical, independent molecules **3** is given for the sake of simplicity. Nonhydrogen atoms are given as probability ellipsoids of atomic displacements (*p* = 0.5).

**Figure 4:**
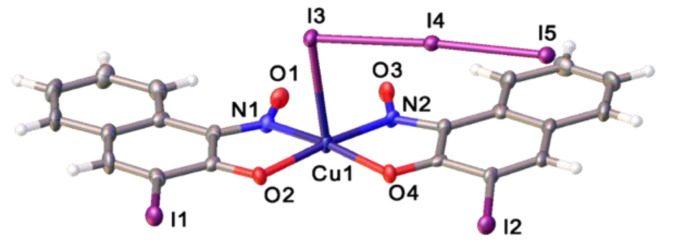
View of the molecular structure of **4** obtained from XRD study. Nonhydrogen atoms are given as probability ellipsoids of atomic displacements (*p* = 0.5).

Crystallization of **2** from the MeOH/THF solution resulted in the release of *trans*-[Cu(I-NON–H)_2_(THF)_2_] (**3**), while the crystallization of *trans*-[Cu(I-NON–H)_2_] from MeOH/Py led to the precipitation of crystals of *trans*-[Cu(Py)_2_(I-NON–H)_2_] (**4**). Complex **1** was crystallized directly from the reaction mixture. Structures of these three complexes were studied by XRD (Figure 2, Figure 3 and Figure 4, Table 2).

The crystal structure of **3** contains two symmetrical, independent molecules of **3**. The structure of **4** consists of independent halves of the complex molecule, while the structure of **1** contains molecules of one type. The structure of **1** demonstrates the *cis*-(*N*,*N*),*cis*-(*O*,*O*) geometry, while **3** and **4** exist in the *trans*-(*N*,*N*),*trans*-(*O*,*O*) form. In **1**, the copper(II) center forms two Cu–N, two Cu–O, and one Cu–I coordination bonds, being in the square-pyramidal environment.

The Cu–I distance (2.9417(8) Å) is slightly longer than those typical for the conventional Cu–I bonds (2.68 Å) [31] and exhibits semicoordinative [32] nature. In **3**, copper(II) is in a distorted octahedral environment, where the equatorial Cu–N (1.964(5)–1.977(5) Å) and Cu–O (1.953(4)–1.957(4) Å) bonds are distinctly shorter than the axial Cu–O_THF_ bonds (2.448(4)–2.535(5) Å). In **4**, the coordination octahedron is less distorted with quite similar distances of the Cu–N (2.018(4) Å) and Cu–N_Py_ (2.028(4) Å) bonds and with a slightly longer Cu–O length (2.302(3) Å). The values of the distances N–O (1.240(6)–1.299(7) Å) and N–C (1.313(8)–1.354(6) Å) of the I-NON–H ligands in **3** and **4** and one I-NON–H ligand in **1** support the existence of these ligands predominantly in the nitrosophenolate form. In contrast, the ligated I-NON in **1** demonstrates the interatomic separations (N–O 1.330(7), N–C 1.313(8), and C–O 1.249(8) Å), which are more typical for the quinone monoxime form.

Several intermolecular noncovalent interactions were identified in the structures of **1** and **3**–**4** (Table 3). In **1**, the Cu center forms the semicoordinative bond with an I atom of the I_3_^–^ ligand of neighboring complex molecule. The Cu1–I4 distance (3.59 Å) is greater than the corresponding sum of Bondi van der Waals radii [33,34] (3.38 Å), but is still substantially smaller than sum of Batsanov crystallographic van der Waals radii (4.1 Å) [35], and, therefore, the Cu1–I4 linkage can be attributed to the semicoordination bond [32].

### 2.3. Halogen Bonding Interactions Involving Ligated I-NON

Organoiodine atoms from I-NON–H and I-NON ligands form HaBs [21] with iodine atoms of the I_3_^−^ ligands. In **3**, I···O HaBs were detected between I-NON–H ligands. In all structures, several intermolecular hydrogen bonds, e.g., H_aryl_···O, H_aryl_···I, were also recognized. In view of the importance of HaB interactions in crystal engineering and to verify s-hole properties of the ligated I-NON, we studied HaBs in the structures of **1** and **3** in more detail by computational methods.

**Figure 5:**
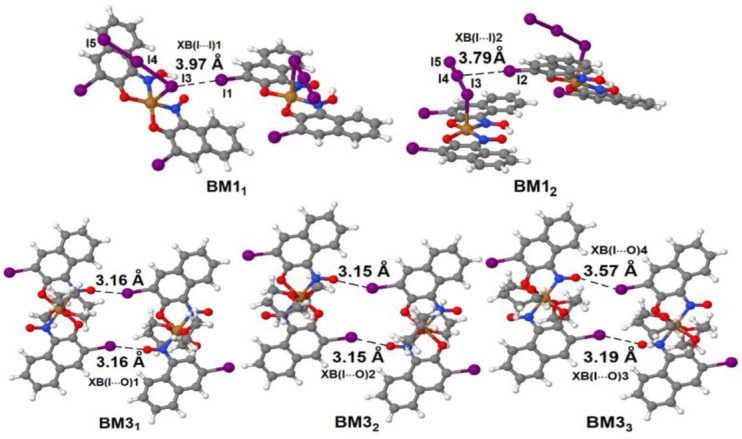
Computational models.

The nature of the HaBs in **1** and **3** was studied theoretically at the DFT (PBE0-D3BJ) level using the quantum theory of atoms in molecules (QTAIM), electrostatic potential (ESP), electron localization function (ELF), natural bond orbital (NBO), and independent gradient model (IGM) analyses. The calculations were carried out for bimolecular clusters **BM1_1_**, **BM1_2_**, **BM3_1_**, **BM3_2_**, and **BM3_3_** with geometries corresponding to the XRD structures of **1** and **3** (Figure 5, see Computational Details). In **1**, two I···I HaBs formed by the organoiodine atoms of the I-NON–H and I-NON ligands were recognized, i.e., HaB(I···I)1 and HaB(I···I)2 (Table 3). The former bond clearly belongs to type II [27] interactions, with the I4–I3···I1 and C–I1···I3 angle being 135.9 and 178.8°, respectively. Classification of HaB(I···I)2 based on geometrical parameters is not so straightforward since the C–I2···I4 angle is 150.6°. However, the ESP, ELF, and NBO analyses clearly indicate that this bond is also of type II with the I2 atom serving as an HaB donor. The low value of the C–I2···I4 angle is accounted for by packing effects, which force the deviation of this HaB from the optimal directionality. In **3**, four I···O bondings were detected, i.e., HaB(I···O)1–HaB(I···O)4 (the C–I···O and N–O···I angles being 138.1–153.0° and 142.7–160.9°, respectively). Geometry of these interactions resembles the halogen···halogen type I [27] interactions.

**Figure 6:**
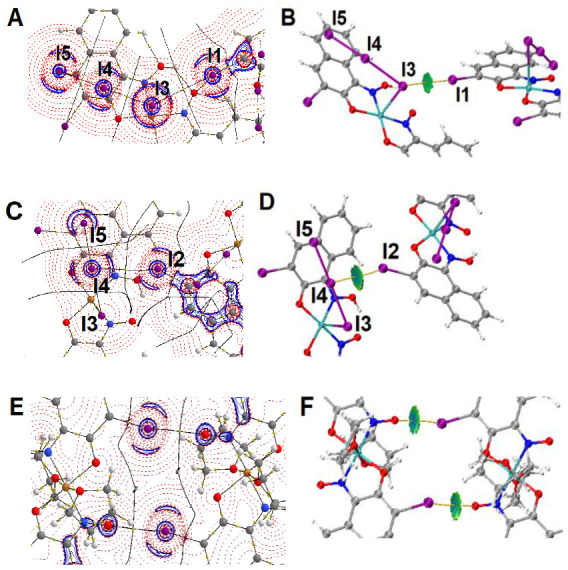
Contour line diagram of the Laplacian distribution ∇^2^ρ(r), selected bond paths, zero flux surfaces (**A**,**C**,**E**) and the sign(λ_2_)ρ(r) function mapped on the δg^inter^ isosurface of 0.005 a.u. and blue-cyan-green-yellow-red color scale −0.02 < sign(λ_2_)ρ(r) < 0.02 for the I···I and I···O contacts (**B**,**D**,**F**) for **BM1_1_** (**A**,**B**), **BM1_2_** (**C**,**D**) and **BM3_1_** (**E**,**F**).

The QTAIM analysis revealed bond critical points (BCPs) for all these contacts (Figure 6). The calculated values of electron density, ρ_b_, its Laplacian, ∇^2^ρ_b_, potential, and kinetic energy densities (V_b_ and G_b_) at the BCP are typical for halogen bonds of weak-to-medium strength [36,37,38,39] (Table 4). These parameters are lower for HaB(I···I)1 and HaB(I···O)4 correlating with the longer I···I and I···O contacts in these bonds. Meanwhile, all these bondings have an attractive nature, as indicated by the negative sign of the second eigenvalue of the Hessian matrix at the BCP, λ_2,b_ (Table 4), and confirmed by the IGM plots of the sign(λ_2_)ρ(r) function mapped on the isosurface of the δg^inter^ descriptor (Figure 6).

**Figure 7:**
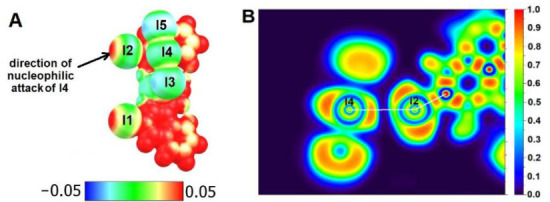
ESP distribution mapped on the 1.1× van der Waals surface for **M1** (**A**) and ELF for **BM1_2_** (**B**).

Formation of type II HaB may be interpreted as a result of the electrostatic interaction between the region of the negative ESP around the HaB acceptor atom and the region of the positive ESP (σ-hole) at the HaB donor atom. Indeed, in the optimized structure of the monomer *cis*-[Cu(I-NON–H)(I-NON)](I_3_) (**M1**), the maximum values of ESP at the terminal ends of the C–I bonds calculated on the 1.1× van der Waals surface are 0.054 and 0.061 a.u. (Figure 7A). Despite the C–I2···I4 angle in **1** is significantly lower than 180°, a belt of the negative ESP around the I4 atom is still directed towards the σ-hole of I2 providing the electrostatic interpretation of this bond. The ELF analysis also demonstrates that the maximum function value for the monosynaptic basin of the I4 atom is directed towards the ELF minimum at the I2 atom (Figure 7B).

The charge transfer (CT) plays only an insignificant role in these HaBs. The NBO analysis indicated that the main contribution in CT in HaB(I···I)1 and HaB(I···I)2 comes from the LP(I3)→σ*(I1–C) and LP(I4)→σ*(I2–C) transitions with the second order perturbation energies E(2) of only 1.3 kcal/mol. Meanwhile, such CT confirmed the attribution of both these interactions to the type II HaBs with the I1 and I2 atoms serving as HaB donors; the back CT was not significant. The charge transfer in HaB(I···O)1–HaB(I···O)3 is even less substantial with E(2) of 0.8–0.9 kcal/mol for the predominant LP(O)→σ*(I–C) transitions and it is negligible for HaB(I···O)4.

The interaction energies of the I···I and I···O bonds were estimated using two approaches (E_int_(BSSE) and E_int_(S); see Computational details). The E_int_ values of the individual bonds are within the range of −4.0 to −10.4 kcal/mol that is typical for HaBs of weak to medium strength, with the I···I bonds being slightly more stable than the I···O ones (Table 4).

## 3. Discussion

We developed a high-yielding facile route toward I-NON: This compound was formed via copper(II)-mediated iodination of NON with I_2_. Among various transition metal salts, copper(II) salts, in particular Cu(OAc)_2_·H_2_O, were found to be the best promotors of this reaction. The metal center played multiple roles in this iodination: Copper(II) stabilized NON in a deprotonated nitrosophenolate form by the coordination, thus preventing oxidative deoximation; the anionic aromatic NON–H^−^ form is preferable for aromatic electrophilic substitution involving such halogen as iodine. In addition, copper(II) activated I_2_ as an electrophilic agent [16]. Unlike Cu^II^-mediated iodination of various aromatics, the NON iodination product was formed in the coordination sphere and, therefore, an additional step of the formed ligand liberation, by the reduction using Na_2_S_2_O_3,_ was required.

We also employed the prepared I-NON as a chelator and four new I-NON-based copper(II) complexes were obtained. The geometry of these complexes depends on the presence of I-NON ligands in deprotonated form and the ligand environment. Thus, complex **1** exhibited the *cis*-(*N*,*N*),*cis*-(*O*,*O*) geometry and complexes **3** and **4** were isolated as the *trans*-(*N*,*N*),*trans*-(*O*,*O*) isomers. The characteristic feature of the structures of **1** and **3** included intermolecular HaBs with the involvement of organoiodine atom of I-NON ligands. Thus, I-NON is a perspective new chelator, in which the presence of an iodine atom leads to the appearance of new structural features of I-NON-based complexes to give, for instance, HaBs in the solid state. Theoretical studies allowed the interpretation of the I···I and I···O short contacts in **1** and **3** as HaBs of weak-to-medium strength.

## 4. Materials and Methods

### 4.1. Materials and Instrumentations

The 1-Nitroso-2-naphthol, copper(II) salts, I_2_, Na_2_S_2_O_3_, and solvents were obtained from a commercial source and used as received. The HRESI mass spectra were obtained on a Bruker micrOTOF spectrometer equipped with an electrospray ionization source and MeOH was employed as the solvent. The instrument was operated in positive ion mode using an *m/z* range of 50–3000. The capillary voltage of the ion source was set at −4500 V (ESI^+^ MS) and the capillary exit ±(70–150) V. In the isotopic pattern, the most intensive peak was reported. Infrared spectra were recorded using a Bruker FTIR TENSOR 27 instrument in KBr pellets. The absorption spectra were recorded on a Shimadzu UV 1800 spectrophotometer in MeCN. The ^1^H and ^13^C{^1^H} NMR spectra were measured on a Bruker Avance III 400 spectrometer at ambient temperature. Residual solvent signals were used as the internal standard. Microanalyses (C, H, N) were carried out on a Euro EA3028-HT instrument. The TGA studies were performed on a NETZSCH TG 209 F1 Libra thermoanalyzer and MnO_2_ powder was used as a standard. The initial weights of the samples were in the range of 1.1–2.3 mg. The experiments were run in an open aluminum crucible in a stream of argon at a heating rate of 10 K/min. The final temperature was 610 °C. Processing of the thermal data was performed with Proteus analysis software. The powder diffraction experiments for **1** were carried out using D2Phaser diffractometer (Bruker), Cu anode, at 30 kV and 10 mA, and CuKα_1+2_ radiation λ_CuKα1_ = 1.54059 Å and λ_CuKα2_ = 1.54443 Å, from 6° to 60° on the 2θ scale at 1 s/step with step size 0.02°.

### 4.2. X-ray Structure Determinations

The XRD experiments were carried out using Oxford Diffraction “Xcalibur” diffractometer with monochromated MoKα radiation. The crystals were thermostated at 100 K throughout the all-experiment time. The structures were solved by ShelXT [40] and Superflip [41] structure solution programs using Intrinsic Phasing and Charge Flipping methods, respectively, and refined using ShelXL [40] minimization program incorporated in Olex2 [42] program package. Empirical absorption correction was accounted by CrysAlisPro (Agilent Technologies, 2013) using spherical harmonics, implemented in SCALE3 ABSPACK scaling algorithm. The crystallographic data were deposited in the Cambridge Crystallographic Data Centre under the deposition codes CCDC 2076962–2076965 and can be obtained free of charge via the Internet, URL http://www.ccdc.cam.ac.uk/structures/ (accessed on 17 September 2021).

### 4.3. Synthetic Work

The **3-iodo-1-nitroso-2-naphthalenol**. Cu(OAc)_2_·H_2_O (28.9 mg, 0.145 mmol), 1-nitroso-2-naphthol (50 mg, 0.289 mmol), and iodine (293.6 mg, 1.16 mmol) were stirred at RT in CHCl_3_/MeOH (1/1 *v*/*v*; 30 mL) overnight. The solvent was then evaporated to dryness, and the residue formed was dissolved in THF (30 mL) and treated with a saturated aqueous solution of Na_2_S_2_O_3_ (25 mL). The organic layer was dried over MgSO_4_ and then the solvent was removed in vacuo at 30–35 °C to give a crude product. Purification by column chromatography (silica gel, hexane/ethyl acetate 4:1, *v*/*v*) afforded I-NON (40.6 mg, 94%) as a dark-brown powder. Alternatively, the product can be purified from NON by sublimation of the latter at 100 °C (0.07 torr).

Yield 40.6 mg, 94%. Dark brown residue. Anal. Calcd for C_10_H_6_NIO_2_: C, 40.16; H, 2.02; N, 4.68. Found: C, 40.01; H, 1.95, N, 4.75%. HRESI^+^-MS, *m/z*: 321.9300 ([M + Na]^+^ requires 321.9341), 620.8707 ([2M + Na]^+^ requires 620.8784). *ν*_max_(KBr)/cм^−1^: 3434, 3355, 3176 m ν(OH); 2922 m, 2852 w ν(C–H); 1668 s ν(C=N or C=O); 1594 w, 1541 s δ(CH_Ar_). ^1^H NMR (400 MHz, CDCl_3_) δ 17.16 (s, 1H, NOH), 8.40 (s, 1H, HC_4_), 8.34 (d, *J* = 8.0 Hz, 1H, HC_8_), 7.58 (t, *J* = 7.5 Hz, 1H, HC_7_), 7.50 (t, *J* = 7.4 Hz, 1H, HC_6_), 7.44 (d, *J* = 7.5 Hz, 1H, HC_5_). ^13^C NMR (101 MHz, CDCl_3_) δ 177.93 (C_2_), 156.62 (C_4_), 143.03 (C_1_), 131.55 (C_7_), 130.97 (C_10_), 130.21 (C_9_), 129.95 (C_6_), 129.08 (C_5_), 123.42 (C_8_), 97.74 (C_3_). ^13^C *ss*-NMR δ 177.34 (C_2_), 157.05 (C_4_), 140.49 (C_1_), 134.24 (C_7_), 132.63 (C_10_), 131.09 (C_9_, C_6_), 130.11 (C_5_), 125.45 (C_8_), 104.24 (C_3_). On heating in a capillary (2°/min), this complex turns black and it decomposes at 154–156 °С. TGA: 158–228 °C (mass loss 22.5%), 228–576 °C (mass loss 37.6%). Crystals suitable for XRD were obtained from the reaction mixture by its slow evaporation.

**[Cu(I-NON–H)(I-NON)](I_3_) (1).** Cu(C_10_H_6_NO_2_)_2_ (0.025 mmol) was added to a solution of I_2_ (0.2 mmol) in CHCl_3_/MeOH (10 mL, 1/1 *v*/*v*) placed in a 20-mL, round-bottomed flask. The reaction mixture was stirred at RT under ultrasonic treatment until the homogenization was complete and then left to stand at RT for slow evaporation. [Cu(I-NON–H)(I-NON)](I_3_) (17 mg, 67%) was released as black crystals after 7–9 d.

Yield 17 mg, 67%. Dark-brown-red crystals. Anal. Calcd for C_20_H_11_CuI_5_N_2_O_4_: C, 23.07; H, 1.06; N, 2.69. Found: C, 23.21; H, 1.19, N, 2.43%. HRESI^+^-MS: *m/z*: 299.9514 ([I-NON + H]^+^ requires 299.9521), 321.9327 ([I-NON + Na]^+^ requires 321.9341), 533.9123 ([Cu(I-NON–H)(NON)]^+^ requires 533.9153), 620.8766 ([2I-NON + Na]^+^ requires 620.8784), 659.8088 ([Cu(I-NON–H)_2_ + H]^+^ requires 659.8104), 681.7916 ([Cu(I-NON–H)_2_ + Na]^+^ requires 681.7924), 1019.6751 ([Cu_2_(I-NON–H)_3_]^+^ requires 1019.6687). HRESI^–^MS, *m/z*: 126.9059 ([I]^–^ requires 126.9045), 297.9354 ([I-NON − H]^−^ requires 297.9365), 380.7113 ([I_3_]^−^ requires 380.7134), 658.7954 ([Cu(I-NON–H)_2_]^−^ requires 658.8026), 956.7297 ([Cu(I-NON–H)_3_]^−^ requires 956.7391). *ν*_max_(KBr)/cм^−1^: 3443 m ν(O–H); 3018 w ν(C_sp2_–H); 1574 m-s and 1537 s ν(C=N, N=O) and δ(CH_Ar_). On heating in a capillary (2°/min), this complex turns black and it decomposes at 189–190 °С; this decomposition is accompanied with iodine elimination. TGA: 181–576 °C (weight loss 62.1%). Crystals suitable for XRD were obtained on slow evaporation of the reaction mixture.

**[Cu(I-NON–H)_2_] (2).** Cu(OAc)_2_·H_2_O (36.1 mg, 0.18 mmol) and 3-iodo-1-nitroso-2-naphthalenol (108 mg, 0.36 mmol) were refluxed in MeOH (30 mL) for 3 h. The formed black precipitate was filtered off, washed with methanol (2 × 10 mL) and diethyl ether (2 × 10 mL), and dried in air at RT to give the product (77 mg, 66%) as a black powder. Crystals suitable for XRD were obtained by slow evaporation of a THF solution at RT.

Yield 77 mg, 66%. Dark residue. Anal. Calcd for C_20_H_10_CuI_2_N_2_O_4_: C, 36.42; H, 1.53; N, 4.25. Found: C, 36.57; H, 1.61, N, 4.32%. HRESI^+^-MS, *m/z*: 533.9138 ([Cu(I-NON–H)(NON)]^+^ requires 533.9153), 555.8969 ([Cu(I-NON–H)(NON-H) + Na]^+^ requires 555.8957), 659.8121 ([M + H]^+^ requires 659.8104), 681.7948 ([M + Na]^+^ requires 681.7924), 1340.5958 ([2M + Na]^+^ requires 1340.5950). *ν*_max_(KBr)/cm^−1^: 3060 w, 2921 w ν(C–H); 1576 m-w and 1531 δ(CH_Ar_), 1511 s δ(CH_Ar_) and ν(N=O). On heating in a capillary (2°/min), this complex turns black and it decomposes at 220–221 °С. TGA: 233–577 °C (weight loss 48.8%). Crystals of **3** suitable for XRD were obtained from solution of **2** in CHCl_3_/THF on its slow evaporation. Crystals of **4** suitable for XRD were obtained from a solution of **2** and excess Py in CHCl_3_/MeOH on its slow evaporation.

### 4.4. Computational Details

The calculations for the QTAIM, ESP, NBO, and IGM analyses were carried out using the crystallographic coordinates at the DFT level of theory with the PBE0 functional [43,44] and the atom-pairwise dispersion correction with the Becke–Johnson damping scheme D3BJ [44,45]. The Gaussian 09 program package [46] was used. Cartesian d and f basis functions (6d, 10f) were used in all calculations. The DZP-DKH basis set for non-iodine atoms [47,48,49,50,51] and the ADZP-DKH basis set for the iodine atoms were applied. The latter basis set was constructed from the DZP–DKH basis set by addition of diffuse functions taken from the ADZP basis set. The Douglas−Kroll−Hess second-order scalar relativistic correction (DKH) [52,53] was applied. An ultrafine integration grid was used for numerical integrations.

Five bimolecular clusters (**BM1_1_**, **BM1_2_**, **BM3_1_**, **BM3_2,_** and **BM3_3_**) with geometries corresponding to the XRD structures of **1** and **3** and triplet spin state were used in the calculations. The C–H bonds were fixed at 1.09 Å and the O–H bonds were fixed at 1.0 Å.

The structure of the monomeric complex **M1** was optimized at the PBE0-D3BJ/DZP(I-ADZP) level and the energies and ESP values were refined at the PBE0-D3BJ/DZP-DKH(I-ADZP-DKH) level.

The topological analyses of the electron density distribution with the help of the AIM method of Bader [54] were performed using the program AIMAll [55] while the IGM analysis [56,57] was performed using the Multiwfn 3.8 [58] and VMD [59] software. The bond orbital nature was analyzed by using the natural bond orbital (NBO) partitioning scheme [60].

The interaction energies of the I···I and I···O bonds were estimated using two approaches. In the first one, the interaction energy [E_int_(BSSE)] was calculated as the difference of total energy of dimer and the sum of the energies of monomers with unrelaxed geometries with the corresponding basis set superposition error (BSSE) correction:

E_int_(BSSE) = (E(dimer) − E(monomer1) − E(monomer2) + BSSE)/n

where *n* = 1 for the I···I bonds and *n* = 2 for the I···O bonds. BSSE was estimated using the counterpoise method [61,62].

In the second approach, the interaction energy [E_int_(S)] was calculated as the energy difference of two dimers:

E_int_(S) = E(dimer2) − E(dimer1)

In dimer1, the HaB donor I atom (I1 or I2) forming the halogen bond was substituted by the hydrogen atom and the length of this C–H bond was fixed at 1.09 Å. In dimer2, the equivalent I atom not forming the HaB was replaced by the H atom (see Figure S1 in Supplementary Information for the structures of dimer1 and dimer2).

## Figures and Tables

**Table 1:** Optimization of the reaction conditions. ^1^.

Entry	Conditions			Product Identification	
	M Salt	M/NON Ratio	I_2_/NON Ratio	I-NON/NON Ratio	Yield of I-NON (%) (^1^H NMR)
1	CuCl_2_·2H_2_O	1/2	1/1	1.15/1	38
2	Cu(SO_4_)_2_·5H_2_O	1/2	1/1	1/1	32
3	Cu(acac)_2_	1/2	1/1	1.7/1	6
4	Сu(NO_3_)_2_·2H_2_O	1/2	1/1	1.14	21
5	Cu(OAc)_2_·H_2_O	1/2	1/1	2/1	18
6	Cu(OAc)_2_·H_2_O	1/2	2/1	3.5/1	30
7	Cu(OAc)_2_·H_2_O	1/2	3/1	16/1	81
8	Cu(OAc)_2_·H_2_O	1/2	4/1	13.5/1	94
9	Cu(OAc)_2_·H_2_O	1/4	1/1	0.04/1	2.6
10	Cu(OAc)_2_·H_2_O	1/4	3/1	2.7/1	53
11	CuI	1/2	4/1	–/1	–
12	Ni(OAc)_2_·4H_2_O	1/2	4/1	0.31/1	<1
13	Co(OAc)_2_·4H_2_O	1/2	4/1	–/1	–
14	FeCl_3_·6H_2_O	1/2	4/1	2/1	23
15	– ^2^	–	2/1	1/1.5	24

^1^ Typical reaction conditions: (1) Metal salt (0.08–0.15 mmol), 1-nitroso-2-naphthol (0.3 mmol), and molecular iodine (0.3–1.2 mmol) in CHCl_3_/MeOH (1/1 *v*/*v*; 30 mL), at RT overnight, whereupon the solvent was evaporated to dryness; (2) The residue formed was dissolved in THF (30 mL) and treated with a saturated aqueous solution of Na_2_S_2_O_3_ (25 mL). Organic layer was dried over MgSO_4_ and then the solvent was removed in vacuo at 30–35 °C to give the crude products, which were analyzed by ^1^H NMR. ^2^ After keeping for 24 h, the system was analyzed by liquid chromatography.

**Table 2:** Selected bond lengths for the Cu^II^ complexes.

Bond	Bond Length, Å		
	1	3	4
Cu–N	1.963(5) 1.955(5)	1.964(5)	2.018(4)
		1.967(5)	
		1.969(5)	
		1.977(5)	
Cu–O	1.954(4) 1.976(4)	1.953(4)	2.302(3)
		1.955(4)	
		1.954(4)	
		1.957(4)	
N–O	1.299(7) 1.330(7)	1.260(5)	1.282(5)
		1.268(5)	
		1.240(6)	
		1.240(6)	
N–C	1.313(8) 1.313(8)	1.339(7)	1.341(5)
		1.343(6)	
		1.354(6)	
		1.348(6)	
O–C	1.280(8) 1.249(8)	1.263(6)	1.236(5)
		1.269(6)	
		1.271(6)	
		1.270(6)	
Cu–O_THF_		2.535(5)	
		2.448(4)	
		2.473(6)	
		2.514(6)	
Cu–N_Py_			2.028(4)
Cu–I	2.9417(8)		

**Table 3:** Selected noncovalent interactions in the Cu^II^ complexes.

Contacting Atoms	Distance, Å (N_c_) ^1^	Angle, °	Contact Type/Name
**1**			
Cu1···I4	3.5935(8) (1.06)	Cu1–I4–I5 88.225(17)	semicoordination
I1···I3	3.9656(6) (1.00)	C3–I1–I3 178.76(17) I1–I3–Cu1 97.06(2)	HaB (I···I)1
I2···I4	3.7872(6) (0.95)	C–I2–I4 150.6(1) I2–I4–I5 101.229(17)	HaB (I···I)2
**3**			
I2···O1	3.159(4) (0.90)	C13–I2–O1 152.33 (15) I2–O1–N1 156.7(3)	(I···O)1
I1···O3	3.146(4) (0.90)	C3–I1–O3 150.64(16) I1–O3–N2 152.2(3)	(I···O)2
I2A···O3A	3.187(4) (0.91)	C13A–I2A–O3A 152.97(12) I2A–O3A–N2A 160.9(3)	(I···O)3
I1A···O1A	3.566(4) (1.02) ^2^	C3A–I1A–O1A 138.07(16) I1A–O1A–N1A 142.7(3)	(I···O)4

^1^ The normalized contact (N_c_) is defined as the ratio between the separation observed in the crystal and the sum of Bondi vdW radii of interacting atoms: Nc = d/**∑**_vdW_; **∑**_vdW_(I + Cu) = 3.38 Å, **∑**_vdW_(I + I) = 3.96 Å, **∑**_vdW_(I + I) = 3.50 Å. ^2^ Despite N_c_ > 1, the results of our calculations support the existence of noncovalent bonding.

**Table 4:** Halogen bond lengths, d (in Å), calculated electron density, ρ_b_, its Laplacian, ∇^2^ρ_b_, potential and kinetic energy densities, V_b_ and G_b_, second eigenvalue of the hessian matrix, λ_2,b_, at BCPs (in a.u.) and interaction energies, E_int_ (in kcal/mol).

Cluster	HaB	d(I…I/O)	ρ_b_	∇^2^ρ_b_	V_b_	G_b_	λ_2,b_	E_int_(BSSE)	E_int_(S)
**BM1_1_**	(I···I)1	3.966	0.0058	0.0191	−0.0022	0.0035	−0.0026	−5.1	−9.0
**BM1_2_**	(I···I)2	3.787	0.0089	0.0290	−0.0039	0.0056	−0.0046	−9.2	−10.4
**BM3_1_**	(I···O)1	3.159	0.0095	0.0458	−0.0063	0.0089	−0.0059	−4.0	−7.2
**BM3_2_**	(I···O)2	3.146	0.0100	0.0471	−0.0065	0.0091	−0.0065	−4.0	−6.9
**BM3_3_**	(I···O)3	3.187	0.0089	0.0430	−0.0057	0.0082	−0.0058	−7.23 ^1^	−7.7
**BM3_3_**	(I···O)4	3.566	0.0046	0.0191	−0.0023	0.0035	−0.0026		−5.4

^1^ Total interaction energy of both I···O bonds in **BM3_3_**.

## Data Availability

Data is contained within the article and Supplementary Materials. CIFs are available on www.ccdc.cam.ac.uk/data_request/cif (accessed on 17 September 2021).

## Supplementary Materials

The following are available online: scheme with tautomeric forms of 1-nitrosonaphthalene-2-ol; characterization of I-NON, **1** and **2**; HRESI-MS, IR, UV-vis absorption, ^1^H and ^13^С{^1^H} NMR spectra, TG and DTG curves, and powder and single-crystal X-ray diffraction data for synthesized species; crystal data and structure refinement for I-NON, **1**, **2**, and **4**; views of the fragment of molecular packing of I-NON, **1**, and **3**, demonstrating noncovalent contacts.
