# Supplementary material for: Copper(II)-Mediated Iodination of 1-Nitroso-2-naphthol
Source: Molecules. 2021 Sep 21;26(18):5708. doi: 10.3390/molecules26185708 (PMC8465374; doi:10.3390/molecules26185708)
Supplement: Supplementary file 1 [file molecules-26-05708-s001.zip › molecules-1389531-supplementary.pdf]

Electronic Supplementary Information

Copper(II)-mediated Iodination of 1-Nitroso-2-naphthol

Zarina M. Efimenko,[1] Anton V. Rozhkov,[1] Vitalii V. Suslonov,[1] Maxim L. Kuznetsov,[1,2] Vadim Yu. Kukushkin,[1,3] and Nadezhda A. Bokach*[1]

[1]Saint Petersburg State University, Universitetskaya Nab. 7/9, 199034 Saint Petersburg, Russian Federation

[2]Centro de Química Estrutural, Instituto Superior Técnico, Universidade de Lisboa, Av. Rovisco Pais, 1049-001 Lisboa, Portugal

[3]Institute of Chemistry and Pharmaceutical Technologies, Altai State University, 656049 Barnaul, Russian Federation

Email: n.bokach@spbu.ru

* Corresponding author

Tautomeric forms of 1-nitrosonaphthalene-2-ol

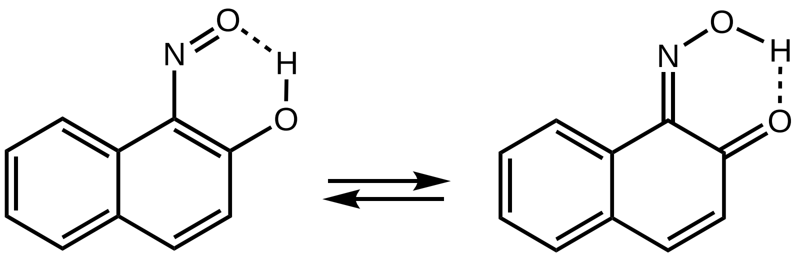

A B

Scheme S1. Two tautomeric forms of NON: 1-nitrosonaphthalene-2-ol (**A**) and 1-(hydroxyimino)naphthalen-2(1*H*)-one (**B**).

Characterization of I-NON, 1 and 2

I-NON was characterized by HRESI^{+}-MS, ^{1}H and ^{13}C{^{1}H} NMR, FTIR, UV-vis spectroscopy, and TG analysis; it gives satisfactory C, H, and N elemental analyses for the proposed formula. The HRESI^{+}-MS exhibits peaks corresponding to [M + Na]$^{+}$ and [2M + Na]$^{+}$. The FTIR spectrum displays the ν(C=N and/or C=O) band at 1668 cm^{-1}, and ν(O–H) bands in the range 3434–3176 cm^{-1}, which are different with those in starting NON (ν(C=N and/or C=O) 1625 and ν(O–H) 3405 cm^{-1} [21]). In the ^{1}H NMR spectrum, the CH_{Ar} protons appear in the interval 8.34–7.44 ppm, while HC_4 emerges as a singlet at 8.40 ppm. The ^{13}C{^{1}H} NMR in $CDCl_3$ and ^{13}C solid-state NMR spectra display acceptable overlap of signals positions, which indicates the presence of I-NON both in a solid state and in a solution in the same *o*-quinonoxime form. The electronic absorption spectrum of I-NON, measured in MeCN, exhibits one band at 270 nm, which correspond to π-π* transitions withing the naphthyl moiety and one broad peak at 394 nm due to the intramolecular charge transfer; the latter band is red shifted relatively to the corresponding band observed for NON (372 nm).

Complexes **1** and **2** characterized by HRESI$^{+/-}$-MS, FTIR, and UV-vis spectroscopy. Both complexes give satisfactory C, H, and N elemental analyses for the proposed formulas. The HRESI^{+}-MS of the complexes exhibit peaks corresponding to [Cu(I-NON–H)$_2$ + H]$^{+}$ and [Cu(I-NON–H)$_2$ + Na]$^{+}$; the HRESI^{-}-MS of *cis*-[Cu(I-NON–H)(I-NON)](I_3) displays peaks of I_3^{-} and

$[Cu(I\text{-}NON\text{–}H)_2]^-$. The FTIR spectra demonstrate intensive bands at 1536 and 1511 cm^{-1} from ν(N=O) for **1** and **2**, correspondingly, and the ν(C=N or C=O) band at 1573 cm^{-1}, and ν(O–H) band at 3444 cm^{-1} for **1**. The absence of any intensive bands in the range 1680–1600 cm^{-1} indicates a significant contribution of the nitrosophenolic form of I-NON–H into the structures of both ligands and the absence (or lower contribution) of the quinone monoxime mesomeric form. Complex **2** demonstrates a greater thermal stability than **1**; the former starts to decompose at ca. 230 °C, while the latter is stable only until 180 °C. The electronic absorption spectra (in MeCN) of both complexes exhibit bands at ca. 230 and 290 nm, which correspond to interligand π-π* transitions, one peak at 421–423 nm due to interligand charge transfer, and low intensity band at 502–503 nm due to metal *d*-*d* transition. Two intensive bands at 299 and 367 nm in the spectrum of **1** were attributed to the interligand I_3^- transitions.

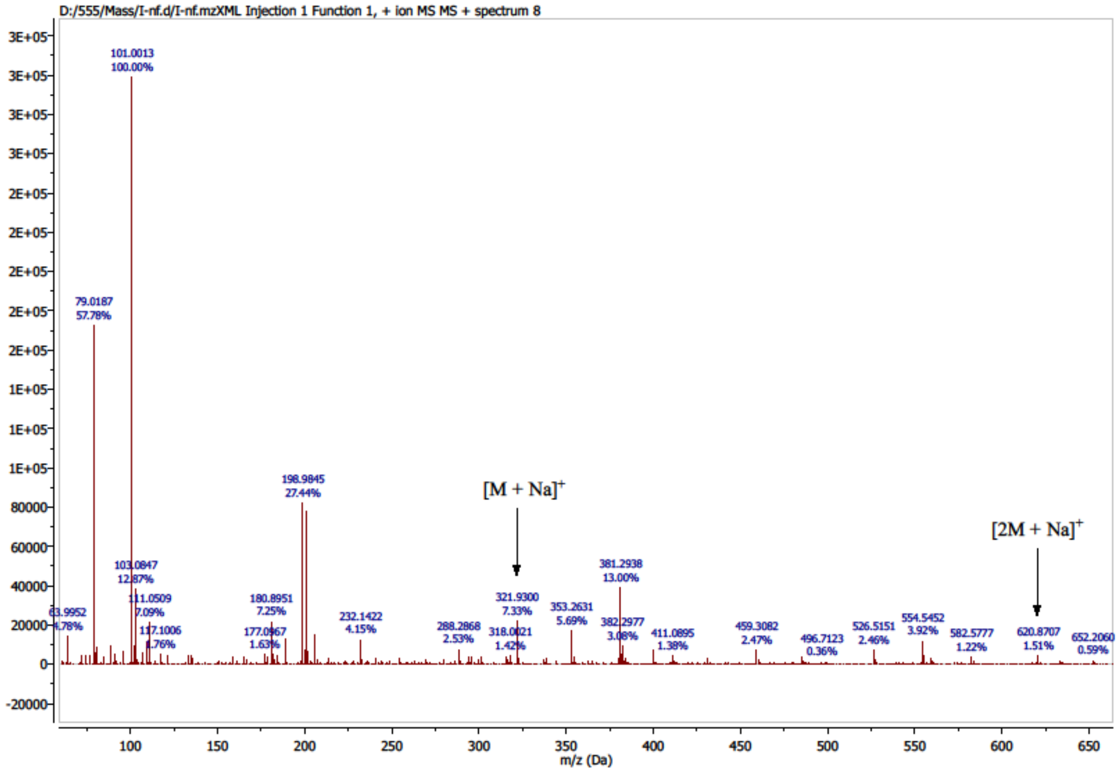

Figure S1. HRESI-MS of I-NON.

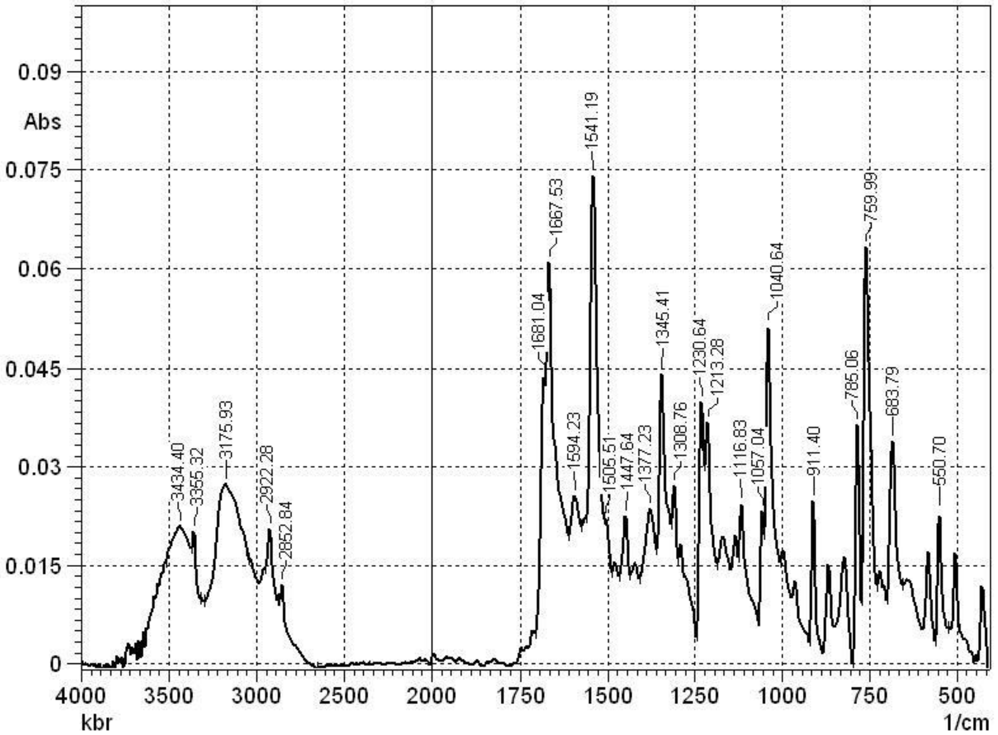

Figure S2. FTIR spectrum of I-NON.

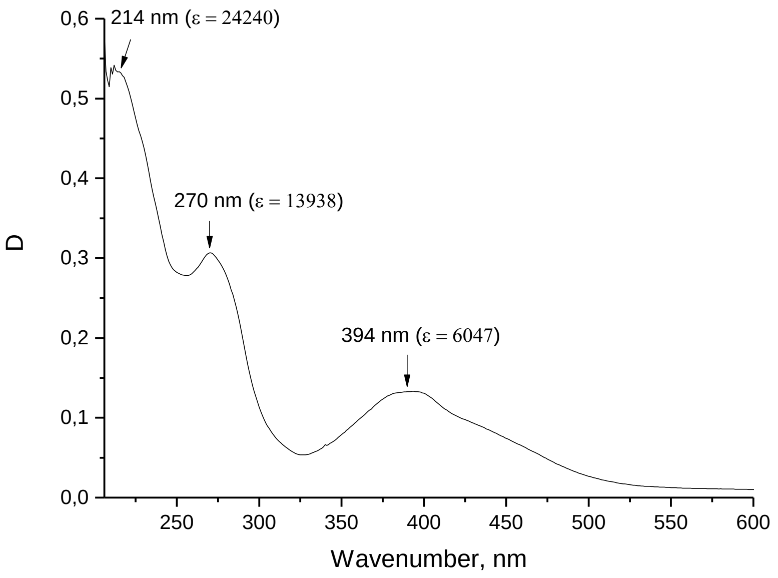

Figure S3. The UV-vis absorption spectrum of I-NON in acetonitrile

(c = 2.2·10^{-4}, l = 0.1).

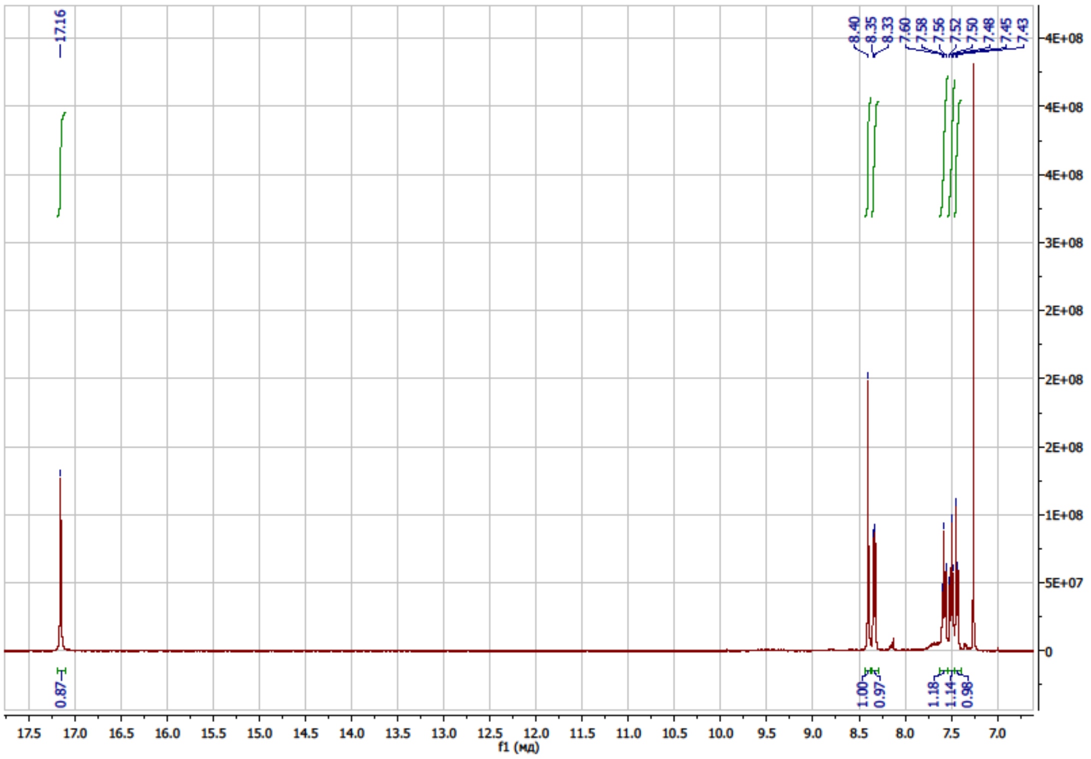

Figure S4. ^{1}H NMR spectrum of I-NON.

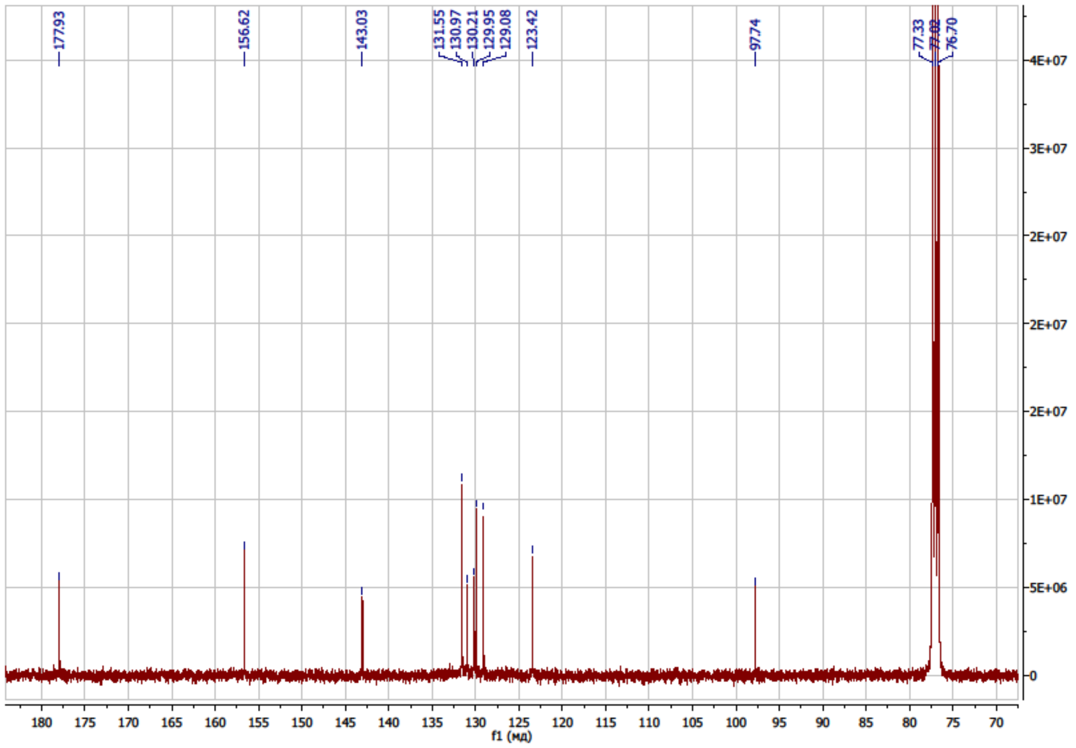

Figure S5. $^{13}C\{^1H\}$ NMR spectrum of I-NON.

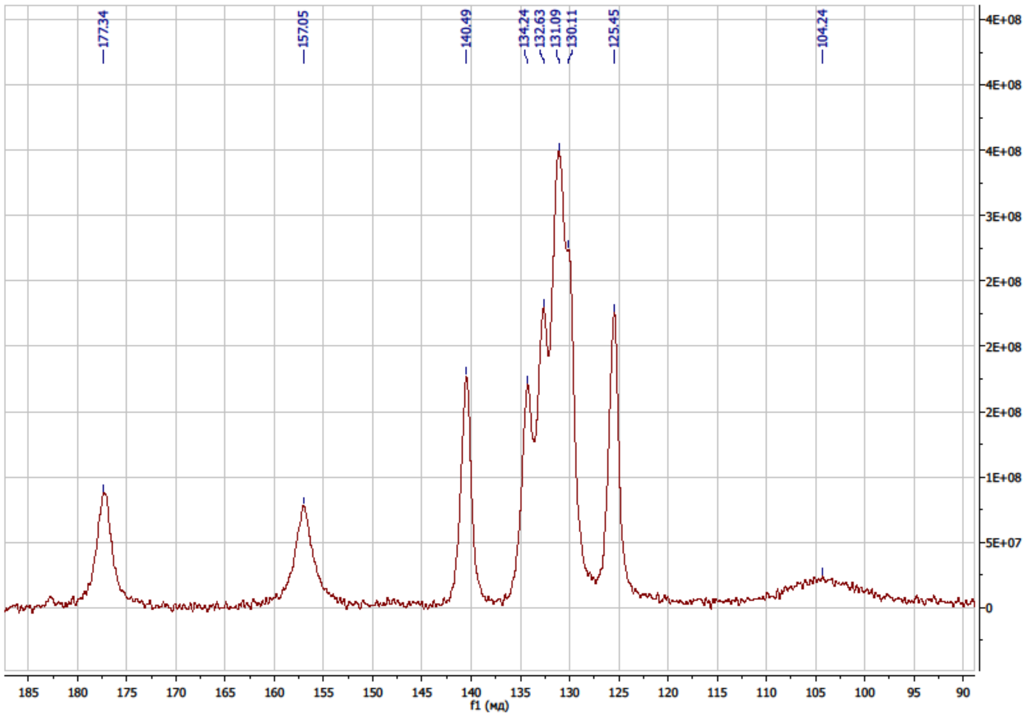

Figure S6. $^{13}C\{^1H\}$ *ss*-NMR spectrum of I-NON.

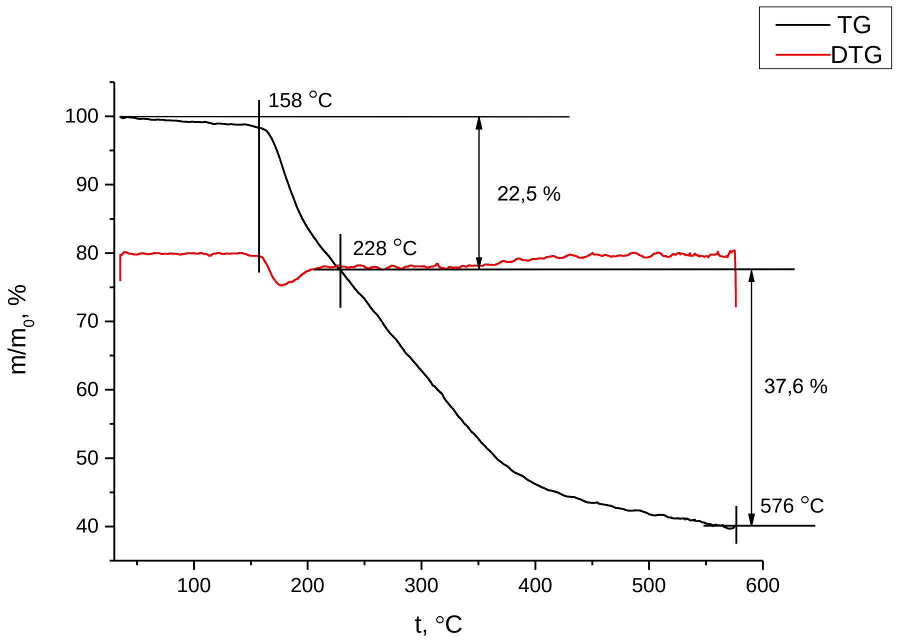

Figure S7. TG and DTG curves for the thermal decomposition of I-NON.

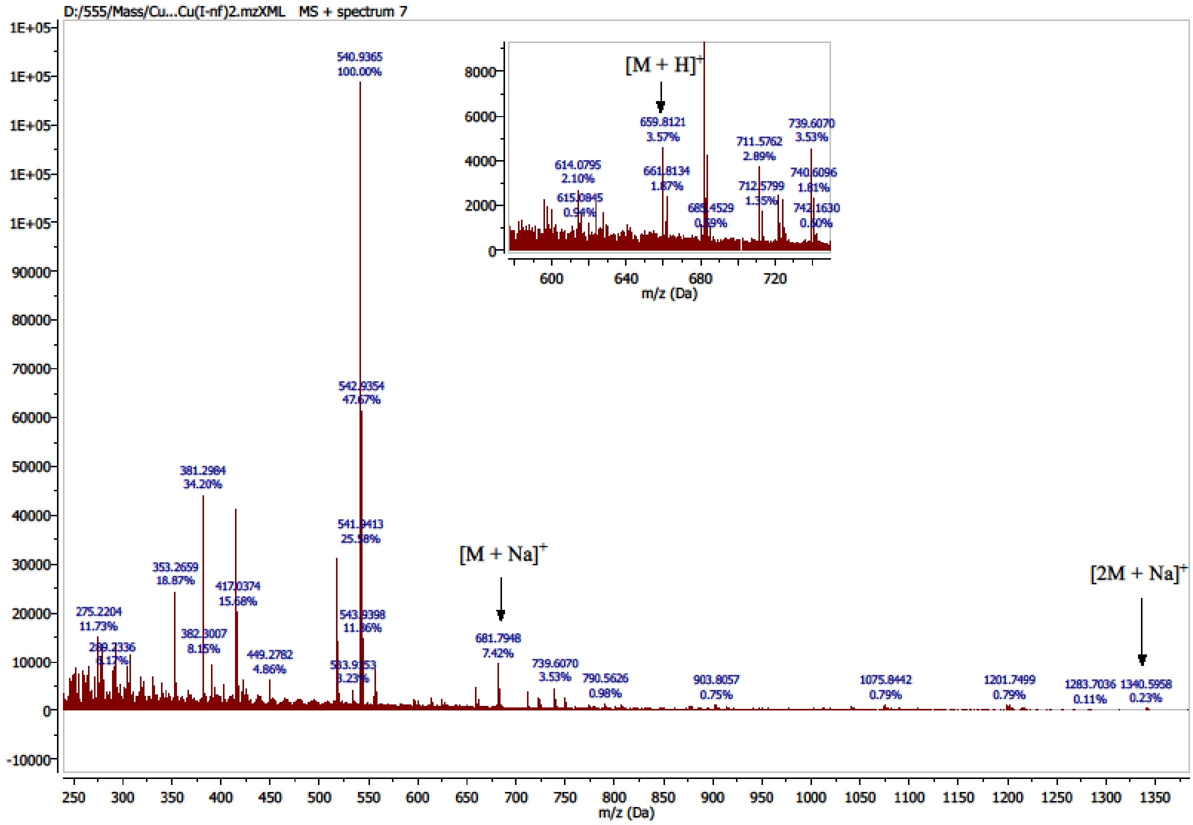

Figure S8. HRESI-MS of **2**.

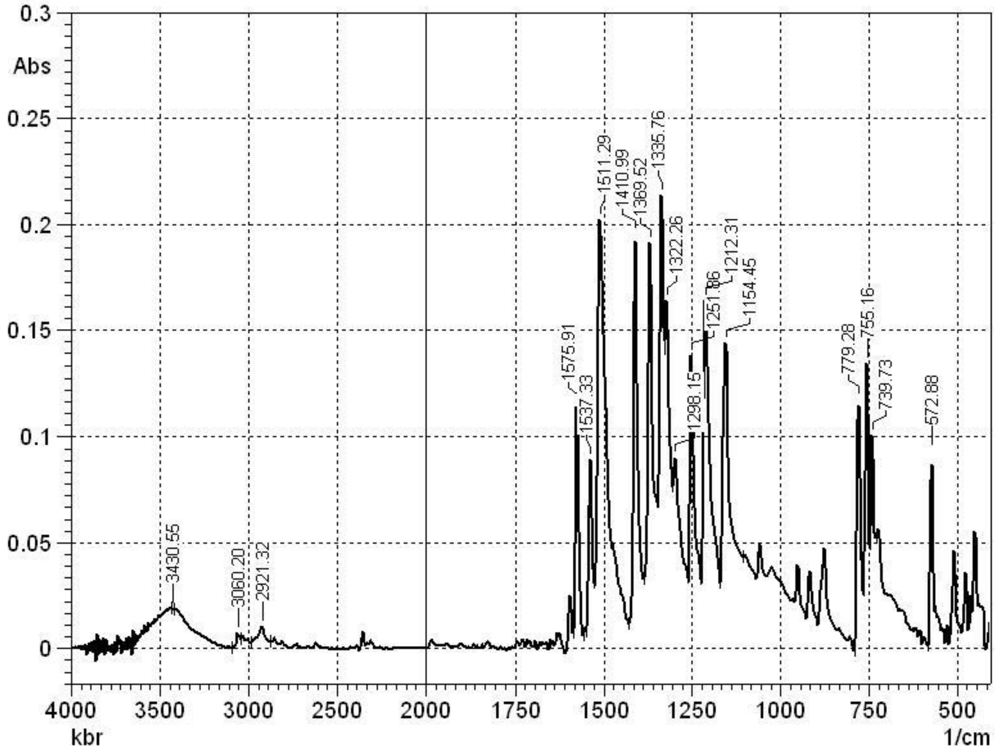

Figure S9. FTIR spectrum of **2**.

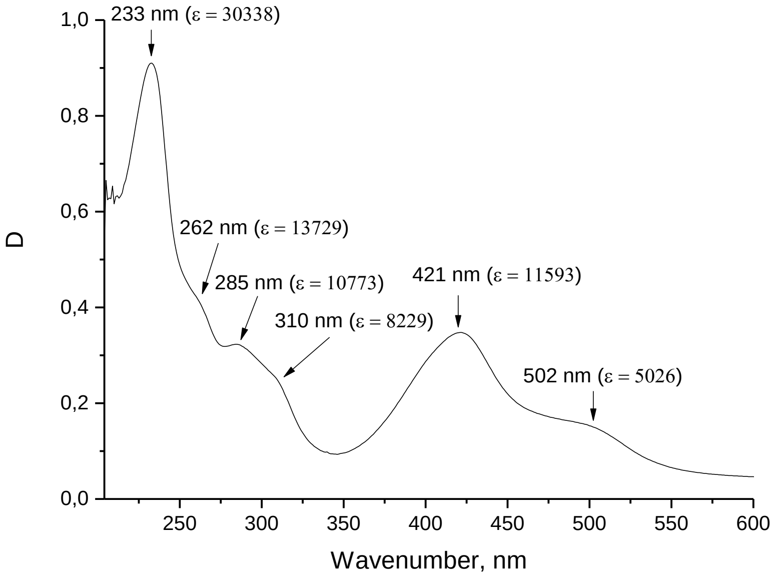

Figure S10. The UV-vis absorption spectrum of **2** in acetonitrile

($c = 3 \cdot 10^{-4}$, $l = 0.1$)

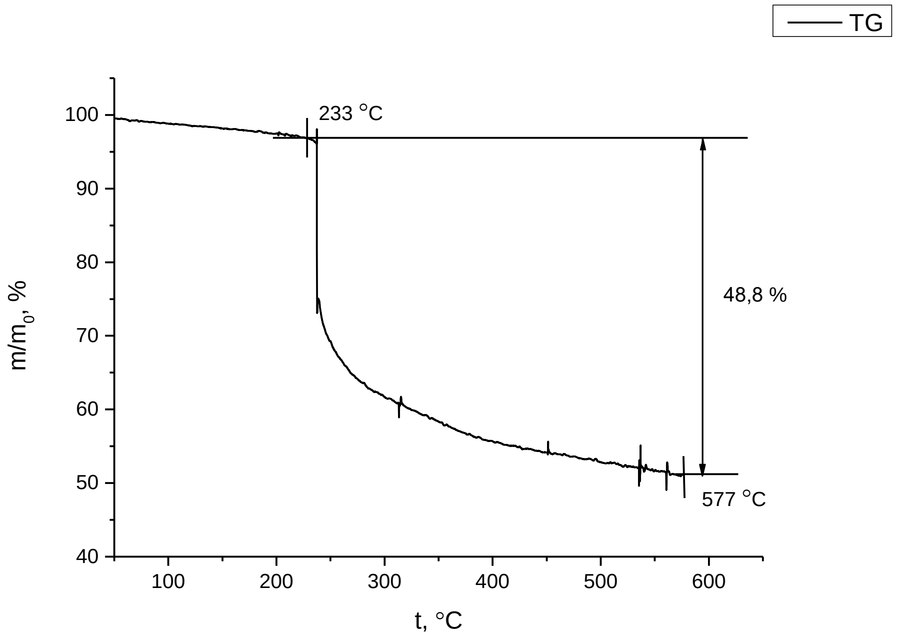

Figure S11. TG curve for the thermal decomposition of **2**.

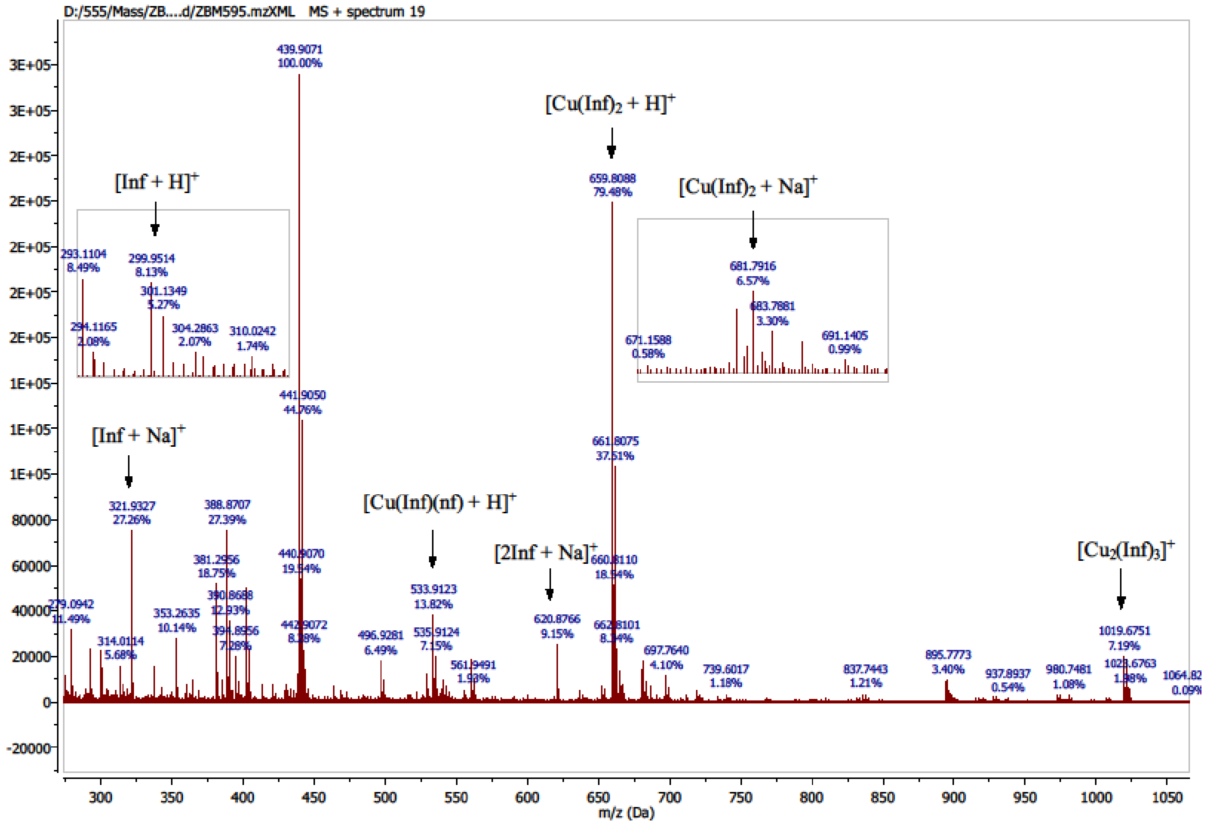

Figure S12. HRESI^{+}-MS of **1**.

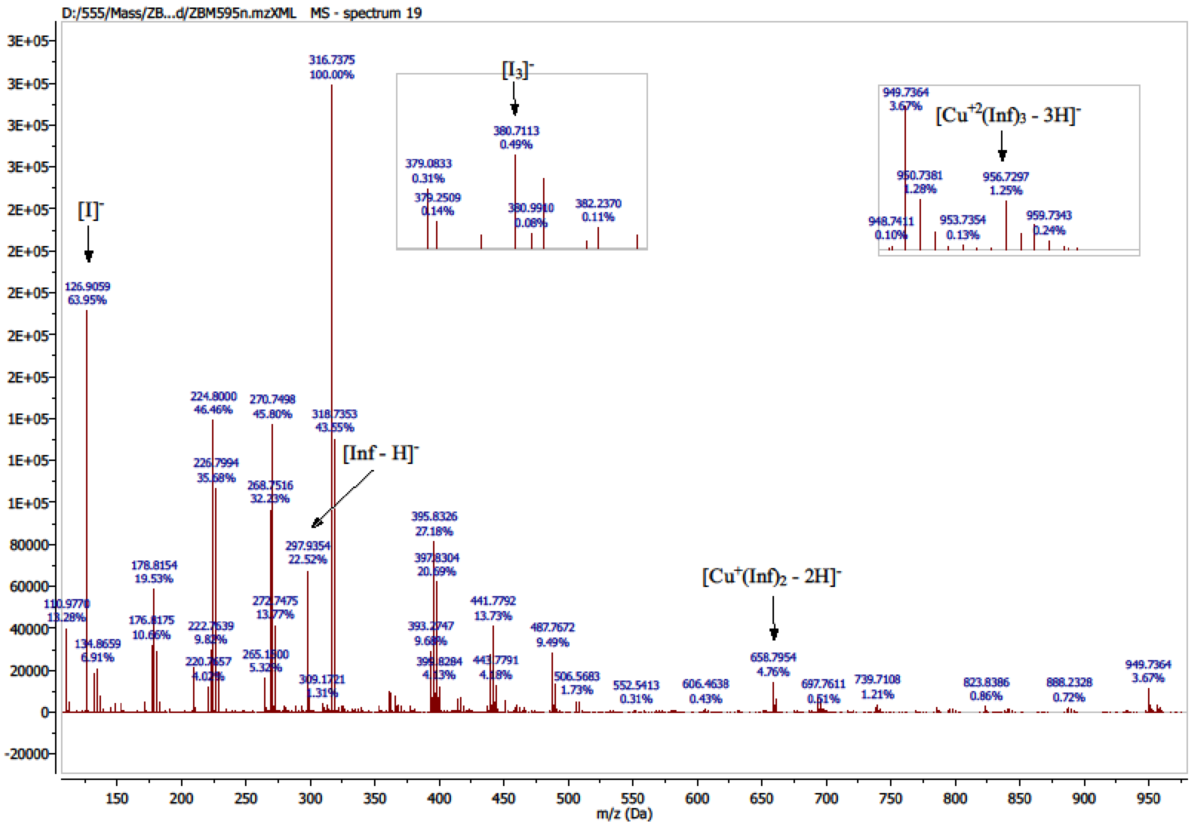

Figure S13. HRESI⁻-MS of **1**.

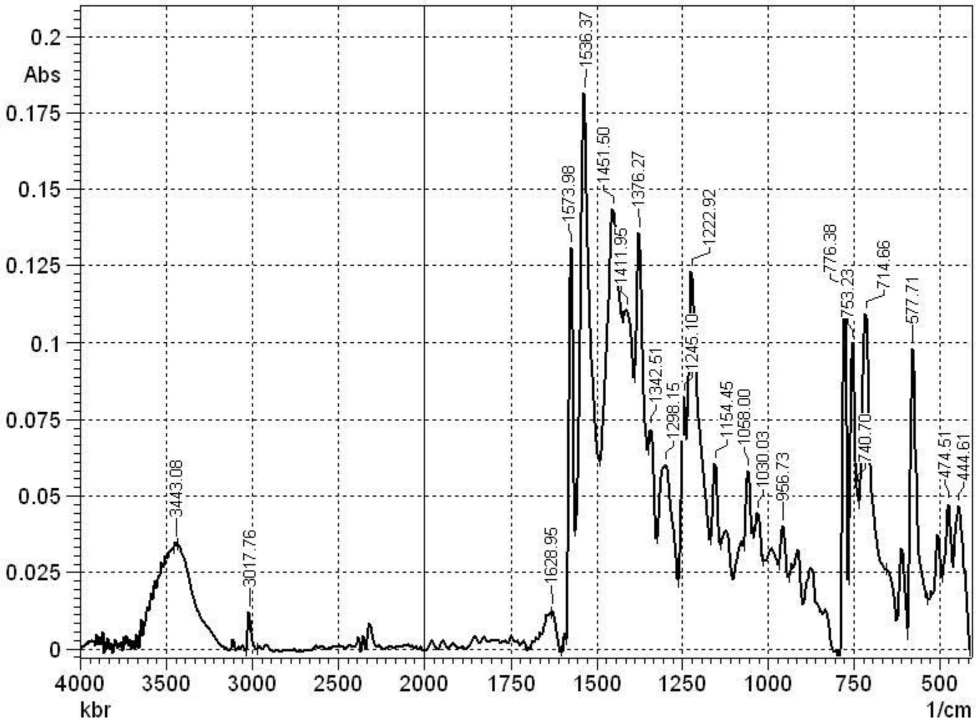

Figure S14. FTIR spectrum of **1**.

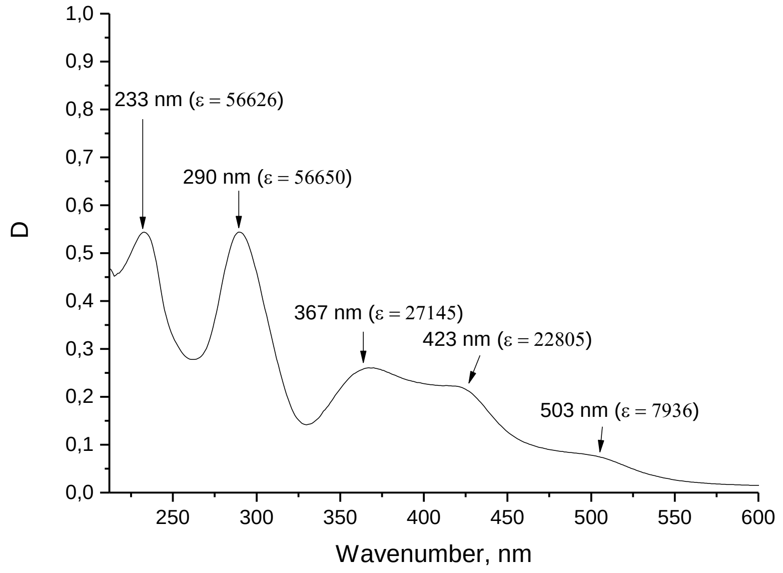

Figure S15. The UV-vis absorption spectrum of **1** in acetonitrile

(c = 9.6·10^{-5}, l = 0.1).

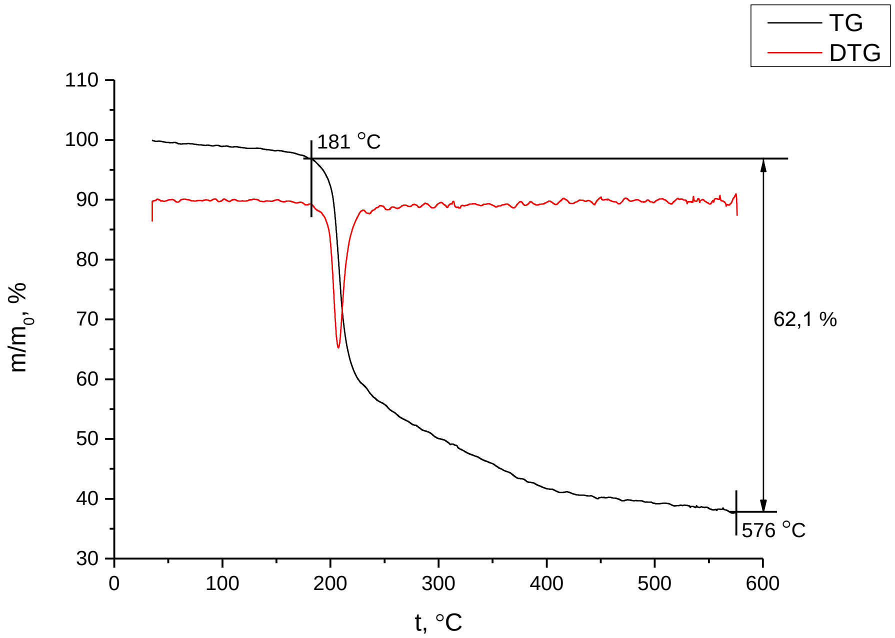

Figure S16. TG and DTG curves for the thermal decomposition of **1**.

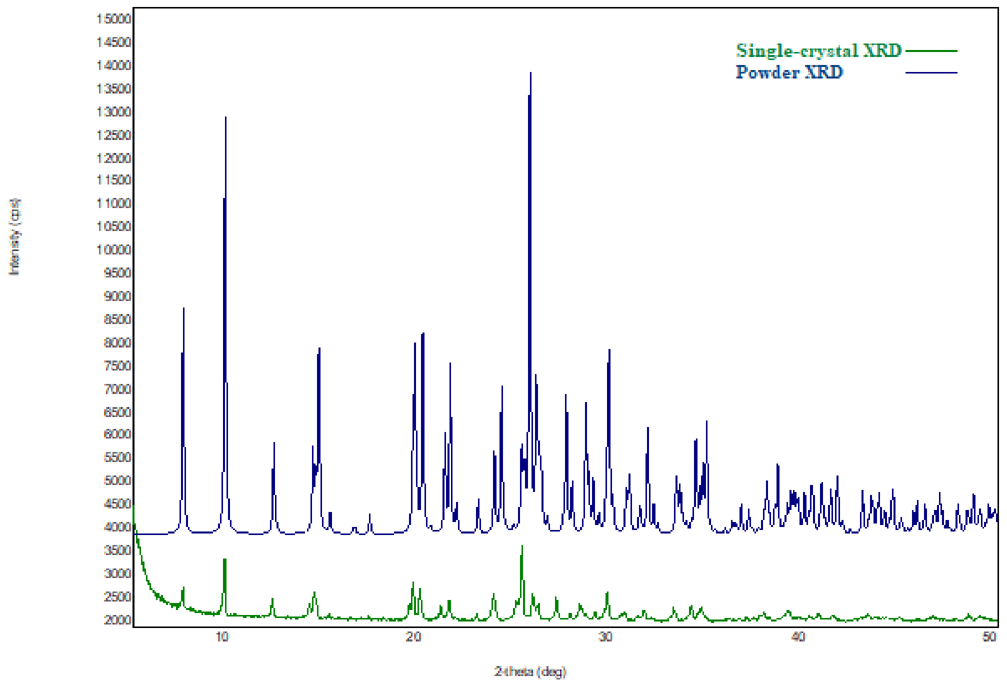

Figure S17. Powder and single-crystal X-ray diffraction data for **1**.

Table S1. Crystal data and structure refinement for I-NON, **1**, **2**, and **4**.

Identification code	**1**	**3**	**4**	I-NON
Empirical formula	$C_{20}H_{10}CuI_5N_2O_4$	$C_{28}H_{26}CuI_2N_2O_6$	$C_{30}H_{20}CuI_2N_4O_4$	$C_{10}H_6INO_2$
Formula weight	1040.34	803.85	817.84	299.06
Temperature/K	100(2)	100(2)	100(2)	100(2)
Crystal system	monoclinic	triclinic	monoclinic	triclinic
Space group	P_n	*P*-1	$P2_1/c$	*P*-1
a/Å	7.43270(10)	9.6702(7)	9.2139(7)	4.1030(7)
b/Å	11.6045(2)	16.8916(8)	17.9586(7)	7.9761(12)
c/Å	14.6496(3)	18.9445(14)	9.0177(5)	14.6239(12)
α/°	90	69.019(6)	90	76.649(10)
β/°	101.497(2)	85.703(6)	110.233(7)	88.025(10)
γ/°	90	77.190(5)	90	78.943(13)
Volume/Å^3	1238.22(4)	2817.4(3)	1400.07(15)	456.98(11)
Z	2	4	2	2
ρ_{calc}g/cm^3	2.790	1.895	1.940	2.173
μ/mm^{-1}	7.145	3.012	3.029	3.472
F(000)	940.0	1564.0	790.0	284.0
Crystal size/mm^3	0.2 × 0.15 × 0.15	0.2 × 0.2 × 0.2	0.2 × 0.2 × 0.15	0.2 × 0.2 × 0.2
Radiation	MoKα (λ = 0.71073)	MoKα (λ = 0.71073)	MoKα (λ = 0.71073)	MoKα (λ = 0.71073)
2Θ range for data collection/°	5.676 to 54.992	4.068 to 55	5.23 to 55.966	5.456 to 54.996
Index ranges	-9 ≤ h ≤ 9, -15 ≤ k ≤ 15, -19 ≤ l ≤ 19	-12 ≤ h ≤ 12, -21 ≤ k ≤ 21, -24 ≤ l ≤ 24	-12 ≤ h ≤ 11, -23 ≤ k ≤ 23, -11 ≤ l ≤ 11	-5 ≤ h ≤ 5, -10 ≤ k ≤ 10, -18 ≤ l ≤ 18
Reflections collected	15005	22802	12008	5718
Independent reflections	5305 [R_{int} = 0.0275, R_{sigma} = 0.0327]	22802 [R_{int} = n/a, R_{sigma} = 0.0898]	3354 [R_{int} = 0.0476, R_{sigma} = 0.0501]	5718 [R_{int} = n/a, R_{sigma} = 0.1120]
Data/restraints/parameters	5305/2/289	22802/0/708	3354/0/187	5718/0/129
Goodness-of-fit on F^2	0.965	0.815	1.142	1.046

Final R indexes [I>=2σ (I)]	R_1 = 0.0198, wR_2 = 0.0408	R_1 = 0.0432, wR_2 = 0.0873	R_1 = 0.0431, wR_2 = 0.0921	R_1 = 0.0671, wR_2 = 0.1458
Final R indexes [all data]	R_1 = 0.0216, wR_2 = 0.0414	R_1 = 0.0866, wR_2 = 0.0937	R_1 = 0.0587, wR_2 = 0.0980	R_1 = 0.0940, wR_2 = 0.1537
Largest diff. peak/hole / e Å^{-3}	0.61/-0.48	1.77/-0.81	1.96/-1.01	2.42/-1.93
Flack parameter	–0.016(18)	n/a	n/a	n/a
CCDC number	2076965	2076963	2076964	2076962

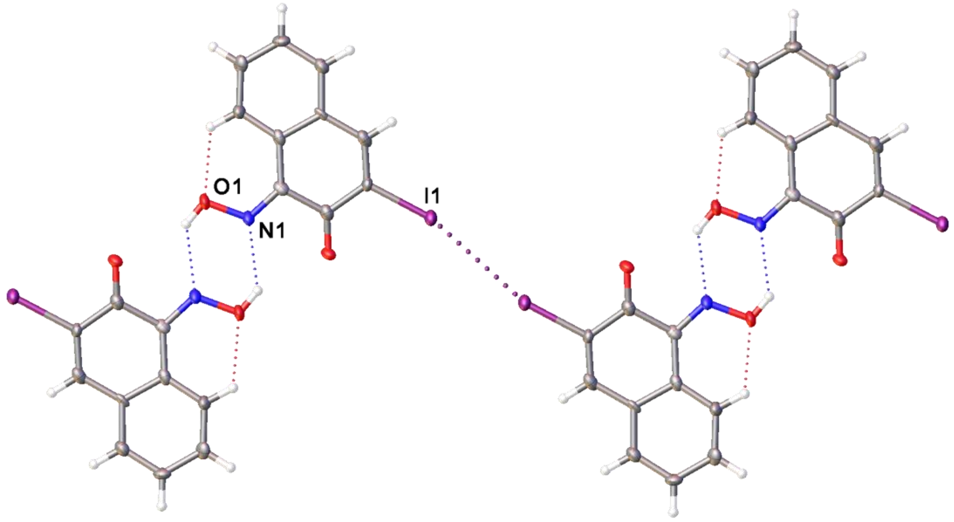

Figure S18. View of the fragment of molecular packing of I-NON, demonstrating intramolecular C–H···O, intermolecular O–H···N HBs and Type I I···I contacts (dotted lines).

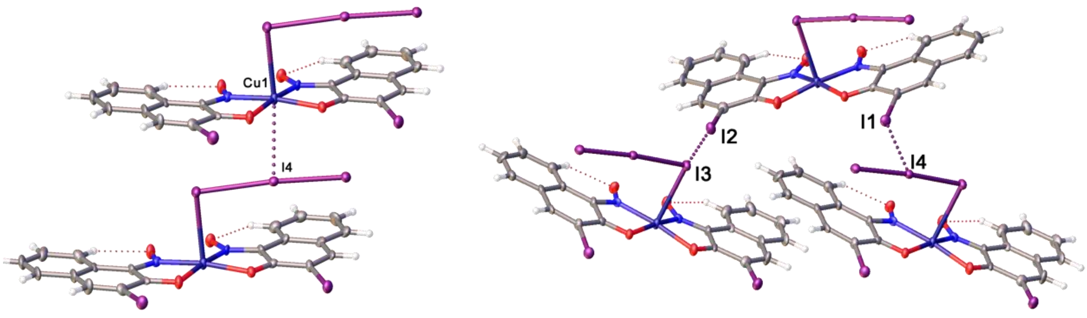

Figure S19. View of the fragment of molecular packing of **1**, demonstrating intermolecular Cu···I semicoordination bond (left panel) and I···I HaBs (right panel).

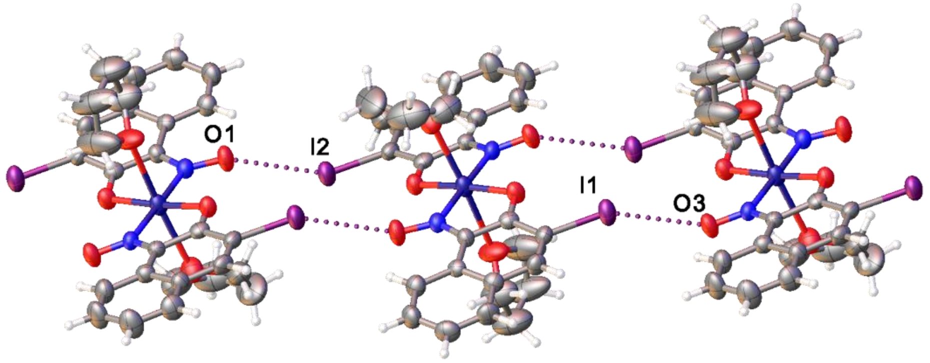

Figure S20. View of the fragment of molecular packing of **3**, demonstrating intermolecular I···O HaBs.
